# Exposure to Alumina Nanoparticles in Female Mice During Pregnancy Induces Neurodevelopmental Toxicity in the Offspring

**DOI:** 10.3389/fphar.2018.00253

**Published:** 2018-03-20

**Authors:** Qinli Zhang, Yong Ding, Kaihong He, Huan Li, Fuping Gao, Taylor J. Moehling, Xiaohong Wu, Jeremy Duncan, Qiao Niu

**Affiliations:** ^1^Department of Occupational Health, School of Public Health, Shanxi Medical University, Taiyuan, China; ^2^Department of Pathology, University of Mississippi Medical Center, Jackson, MS, United States; ^3^Key Laboratory for Biological Effects of Nanomaterials and Nanosafety, Institute of High Energy Physics, Chinese Academy of Sciences, Beijing, China; ^4^Department of Physiology, University of Mississippi Medical Center, Jackson, MS, United States

**Keywords:** alumina nanoparticles, developmental neurotoxicity, neurodevelopmental toxicity, oxidative stress, neurotransmitter

## Abstract

Alumina nanoparticles (AlNP) have been shown to accumulate in organs and penetrate biological barriers which lead to toxic effects in many organ systems. However, it is not known whether AlNP exposure to female mice during pregnancy can affect the development of the central nervous system or induce neurodevelopmental toxicity in the offspring. The present study aims to examine the effect of AlNP on neurodevelopment and associated underlying mechanism. ICR strain adult female mice were randomly divided into four groups, which were treated with normal saline (control), 10 μm particle size of alumina (bulk-Al), and 50 and 13 nm AlNP during entire pregnancy period. Aluminum contents in the hippocampus of newborns were measured and neurodevelopmental behaviors were tracked in the offspring from birth to 1 month of age. Furthermore, oxidative stress and neurotransmitter levels were measured in the cerebral cortex of the adolescents. Our results showed that aluminum contents in the hippocampus of newborns in AlNP-treated groups were significantly higher than those in bulk-Al and controls. Moreover, the offspring delivered by AlNP-treated female mice displayed stunted neurodevelopmental behaviors. Finally, the offspring of AlNP-treated mice demonstrated significantly increased anxiety-like behavior with impaired learning and memory performance at 1 month of age. The underlying mechanism could be related to increased oxidative stress and decreased neurotransmitter levels in the cerebral cortex. We therefore conclude that AlNP exposure of female mice during pregnancy can induce neurodevelopmental toxicity in offspring.

## Introduction

Neurodevelopmental disabilities affect millions of children worldwide (Grandjean and Landrigan, 2014); it is mainly because the placenta does not block the transmission of many environmental toxicants from the maternal to the fetal circulation (Needham et al., 2011). It is known that nanoparticles (NPs), unlike bulk materials, are able to pass through biological barriers, such as the blood–brain barrier (BBB), blood–testis barrier, and placental barrier (Keelan, 2011; Zoroddu et al., 2014; Bakand and Hayes, 2016; Muoth et al., 2016). Therefore, NPs can localize in various organs and tissues and generate toxicological effects (Kagan et al., 2005; Nel et al., 2006; Oesterling et al., 2008; Almeida et al., 2011). Although the safety issues are of high importance, related toxicological studies are far behind the development of nanotechnologies (Chen et al., 2008). Alumina nanoparticles (AlNP) have unique physical and chemical characteristics compared to bulk alumina (bulk-Al); they are widely used in industries of pharmacy, water treatment, and manufacturing (Kagan et al., 2005), raising concern of the biological safety regarding public health.

Studies have shown that AlNP can penetrate biological barriers and accumulate in multiple organs (Chen et al., 2008; Oesterling et al., 2008; Almeida et al., 2011; Morsy et al., 2016b), leading to neurotoxicity (Oesterling et al., 2008; Dong et al., 2011; Zhang et al., 2011; Chen et al., 2013; Shah et al., 2015; Morsy et al., 2016a), immunotoxicity (Braydich-Stolle et al., 2010), genotoxicity (Balasubramanyam et al., 2009a,b), as well as toxicity in the lung (Balasubramanyam et al., 2009a; Li et al., 2016), liver (Shrivastava et al., 2014; Morsy et al., 2016a), kidney (Morsy et al., 2016a), and skin (Dey et al., 2008). Our previous work indicated that AlNP can cause neurotoxicity both *in vivo* and *in vitro* (Zhang et al., 2011, 2013). However, no studies have addressed whether AlNP induces developmental toxicity, and in particular, neurodevelopmental toxicity.

Previous research on metal oxidative NPs found developmental toxicity induced by nano-silver (Xin et al., 2015; Charehsaz et al., 2016; Ema et al., 2017; Pecoraro et al., 2017b), nano-silica (Yi et al., 2016), graphene oxide (Buccheri et al., 2016; Pecoraro et al., 2017a), zinc oxide (Bonfanti et al., 2015; Zhou et al., 2015; Lee et al., 2016; Spence et al., 2016), iron oxide (Di Bona et al., 2015), copper oxide (Maisano et al., 2015; Ganesan et al., 2016; Torres-Duarte et al., 2016), magnesium oxide (Ghobadian et al., 2015), titanium dioxide (Scuderi et al., 2014; Parivar et al., 2015; Rollerova et al., 2015; Brundo et al., 2016), and cerium oxide (Andersen et al., 2016). Moreover, studies have shown that bulk-Al, once entering the brain, persists for a very long time with a half-life ranging from 20% of the lifespan to greater than the entire lifespan (Yokel, 2000, 2002; Priest, 2004; Exley and House, 2011). Due to the continuing accumulation in brain, bulk-Al compound can lead to both neurotoxicity (Nel et al., 2006) and developmental toxicity (Poirier et al., 2011; Abu-Taweel et al., 2012). In particular, bulk-Al can incorporate into the brain of the fetus following gestational exposure of female mice (Cranmer et al., 1986; Yumoto et al., 2001). Since AlNP belong to the group of metal oxidative NPs and have Al compound as its basic chemical component, we hypothesized that AlNP could induce neurodevelopmental toxicity.

Given the high susceptibility of the central nervous system (CNS) to external insults during the developmental period, we address specific concerns on neurodevelopmental toxicity effects of AlNP in the present study. Female mice were treated with AlNP at 13 or 50 nm particle sizes via nasal drip delivery prior to and throughout the entire pregnancy. Physical developmental and motor maturation levels of the pups were tracked as indicators of neurotoxicity, anxiety, and cognitive performance of adolescents and were used to confirm the neurotoxic effect; the underlying mechanism was explored as well. To our best knowledge, this study is the first investigation regarding neurodevelopmental toxicity of AlNP and warrants further studies to address potential neurodevelopmental toxicity associated with NPs.

## Materials and Methods

### Characterization of AlNP

Bulk-Al (10 μm particle size) and AlNP (13 and 50 nm particle sizes) were all purchased from Sigma-Aldrich Corporation (St. Louis, MO, United States). NP stock suspensions were freshly prepared in double distilled (dd) water. Ultrasonic vibration (100 W, 30 kHz) was performed for 30 min before use. AlNP were characterized using a transmission electron microscope (TEM) and the diameters were analyzed by Image-pro plus software. Zeta potential of AlNP was analyzed with a Nano Zetasizer (Malvern Instrument, Ltd., Malvern, United Kingdom).

For the TEM, a droplet of AlNP suspension was placed on a 200-mesh Cu-lacy carbon TEM grid (EM Sciences, Hatfield, PA, United States), and was allowed to dry overnight in a vacuum oven at room temperature. After evaporation, the AlNP were dispersed on the TEM grid. Observation and imaging were performed using a JEOL 2010f instrument (JEOL, Tokyo, Japan) with an operating voltage of 200 kV.

Zeta potential of NPs was measured by a Zetasizer (Malvern Instruments, Ltd.) at 25°C. NP stock was diluted 1:4 in double distilled water and measured in triplicates with 20 sub-runs. The samples were stored for 2 h to achieve equilibrium, and put into an ultrasound bath to disperse any aggregates before the zeta potential measurements.

### Experimental Animals and Treatments

**FIGURE 1:**
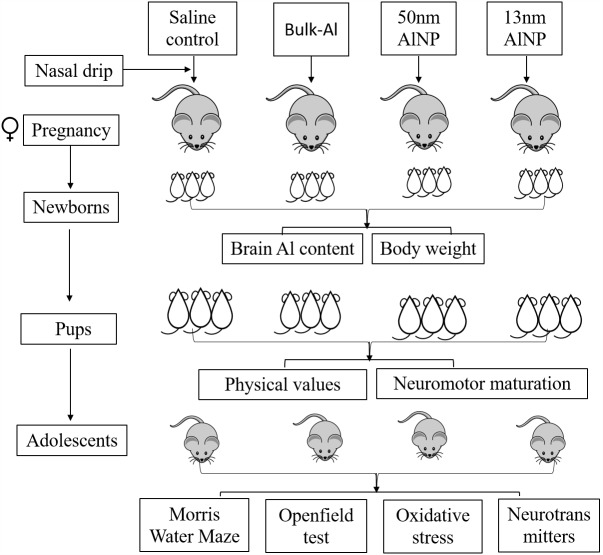
Flowchart of experimental design. Twenty-four female mice were randomly divided into four groups and were treated with saline control, bulk-Al, or AlNP (50 or 13 nm particle sizes) during pregnancy, respectively. Aluminum content and body weight were measured in newborns, neuromotor maturation tests were tracked in pups, and Openfield and Morris water maze task were performed in adolescents followed by oxidative stress and neurotransmitter levels measurement.

Thirty-six adult ICR mice (25–30 g, 12 males and 24 females) were purchased from Chinese Science Academy Animal Research Institute, and acclimatized for 1 week before the start of the experiment. The animal experiments were performed in accordance with the “Policies on the Use of Animals and Humans in Neuroscience Research” published by the *Society for Neuroscience* in 1995. The protocol was approved by the Animal Administration and Ethics Committee of Shanxi Medical University. All the mice were fed with homemade standard laboratory food (Shanxi Medical University, China) and water *ad libitum* at (23 ± 3)°C, relative humidity of (50 ± 10)%, a 12 h light/dark cycle, and ventilation of (10–20) times/h. Temperature and relative humidity were monitored daily. After acclimation, 24 female mice were randomly divided into four groups, six for each group, which were treated with 25–35 μL of saline control, bulk-Al, or AlNP (50 or 13 nm particle sizes) by nasal drip three times/day at the concentration of 50 mg/kg of body weight. The female mice were mated with healthy untreated male mice overnight after a 2-week treatment period. The male mice were removed after successful mating and the female mice were continually exposed to the treatments until birth of offspring. Pups were randomly chosen from each litter group for tests, two pups for detection of aluminum content, eight pups for body weight and neuromotor maturation tests, and two adolescents (one male and one female) for Openfield and Morris water maze (MWM) task. In total, 12 pups from each treatment group were used for aluminum content measurement, 48 pups for body weight and neuromotor maturation tests, and 12 adolescents (six males and six females) for Openfield and MWM task followed by oxidative stress and neurotransmitter levels’ measurement (**Figure 1**).

### Body Weight

Body weight (BW) used as an indicator of development was tracked in newborn pups every 3 days from postnatal day (PD) 1 through PD28.

### Aluminum Content in the Hippocampus of Newborns

Newborns were anesthetized with 75% ice-cold ethanol for 2 min. Next, brains were carefully removed, and the hippocampus was dissected on an ice-cold glass tube. Then, the hippocampus was digested with a multi-wave 3000 sample preparation system (PerkinElmer, Inc., Shelton, WA, United States) according to the manufacturer’s instruction. In the presence of 5% HNO_3_, aluminum content of samples was analyzed using graphite furnace atomic absorption spectrometry as previously described with minor modifications (Gorsky and Dietz, 1978).

### Physical Assessment During Development

Physical developmental landmarks are morphological indicators of development. Examples of such landmarks, including ear pinna open, eye opening, and teeth appearance, were observed in the developing offspring starting from PD1 through the entire developing period until PD28. The exact day in which the neonatal ear pinna completely detached from the head, both eyes opened, and the emergence of teeth through the gum line was recorded.

### Assessment of Neuromotor Maturation During Developing Period

To assess neuromotor maturation and general behavioral profiles in offspring of AlNP-treated female mice, a routine testing battery was performed on each pup.

#### Righting Reflex

A pup was placed in a supine position on a rough horizontal board and righting reflex was determined to be present if the pup was able to flip itself to the normal dorso-ventral position within 2 min. The test was performed twice for each pup on both PD4 and PD7.

#### Cliff Avoidance

A pup was placed on a platform (30 cm height) with its forepaws touching the edge and head facing outwards from the center of the platform. Cliff avoidance was considered positive if the pup moved its body away from the “cliff” within a 1 min.

#### Endurance Test

It is also referred to as the forelimb grip strength test. Endurance was measured on PD12 and PD14. The forelimbs of pups were positioned on a wooden pole with a diameter of 0.5 cm. The amount of time that the pup was able to grasp the pole was recorded.

### Behavioral Tests for Adolescents

The Openfield and MWM tests were used to evaluate anxiety and cognitive performance in adolescent mice.

#### Openfield Test

The Openfield test was conducted on PD28. General motion in a grid was measured in an Openfield apparatus, consisting of a square wooden arena with surrounding walls (80 cm × 80 cm × 30 cm). The floor of the arena was divided into 64 squares of equal size. The visual observations in the arena lasted 300 s for each mouse. The path of mouse was recorded by a video camera (Sony CCD-IRIS model) that was placed above the arena and was connected to a VHS videocassette recorder (Panasonic AG-5700 model). The video tracking program Etho-Vision (Noldus Information Technologies, Wageningen, Netherlands) was used to record the total distance traveled in 16 central grids and the total duration that the mouse stayed in the center grids. If the mouse reared (forelimbs upright against the wall) or groomed for longer than 3 s, these behaviors were also recorded.

#### Morris Water Maze Task

Learning and memory performance of the adolescent mice were tested using MWM task. The water maze consists of a galvanized white circular water tank (90 cm diameter, 50 cm height) filled with clear tap water (22 ± 1°C) to a depth of 15 cm. A 6 cm × 6 cm invisible escape platform was placed 1 cm below the water level and 13 cm from the rim. The water was made opaque by adding white non-toxic paint. The MWM task was performed as previously described (Zhang et al., 2014). Briefly, each mouse underwent daily sessions of four trials for four consecutive days. For each trial, the mouse was released into the water facing the wall at one of the four standard start locations selected at random (N, S, W, or E). When it succeeded in locating the platform, the mouse was allowed to remain on the platform for 15 s. If the mouse failed to find the platform within 60 s, it was assisted by the experimenter and allowed to remain on the platform for the same amount of time. The interval between trials was 20 min. A probe trial was performed 24 h after the last training session. For the probe trial, the platform was removed from the tank and the mouse was allowed to swim freely for 60 s. The experimenters conducted the trials while blind to the group assignments. All of the trials were videotaped using a camera located 2 m above the water surface. The time required to locate the hidden platform in the training trial (latency) and the time spent in as well as the number of entries into the target quadrant during the probe trial were recorded.

### Oxidative Stress Levels in Cerebral Cortex of Adolescents

Superoxide dismutase (SOD) and malondialdehyde (MDA) were measured to assess oxidative stress and damage. Six samples of cerebral cortex were dissected from adolescents 24 h after the last behavioral test. Tissue samples were homogenized in 100× volumes of ice-cold 0.1 mol/L phosphate buffer solution (containing 300 mmol/L sucrose and 0.1 mmol/L ethylenediamine tetra acetic acid and adjusted to pH 7.8) to yield a 1% (w/v) homogenate. This was then centrifuged at 4°C at 10,000 ×*g* for 10 min. Protein concentration was determined by the dye binding in a Bradford assay using bovine serum albumin as a standard. SOD and MDA contents were assayed using commercially available kits according to the manufacturer’s protocol (Nanjing Jiancheng Bioengineering Institute, China).

### Neurotransmitter Enzyme Activity

Choline acetyltransferase (ChAT) and total cholinesterase (TChE) were assessed as potential mechanism underlying cognitive dysfunction in adolescent mice, since ChAT and TChE have been shown to be directly associated with acetylcholinergic content in rodents (Tayebati et al., 2004). Biochemical tests for cholinergic enzymes were conducted in adolescents 24 h after the last behavioral test. Half of the cerebral cortex from each mouse was dissected and homogenized with ice-cold 150 mM KCl. The homogenate (10% w/v) was centrifuged at 10,000 ×*g* for 15 min, and the supernatant was used for analysis. TChE activity was measured by the modified Ellman’s colorimetric method (Ellman et al., 1961; Ingkaninan et al., 2000), and ChAT activity was measured using the chemiluminescence assay described previously (Vijayaraghavan et al., 2013).

### Statistical Analysis

For quantitative data, the data are expressed as mean ± standard deviation (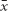 ± s). One-way analysis of variance (ANOVA) is used to test the difference between means of the four subgroups of a variable (multiple testing) following by Dunnett’s *post hoc* test to evaluate the statistical difference between the experimental groups and the controls, and Student–Newman–Keuls (SNK) test to assess the difference between experimental groups. For categorical data, the data are expressed as numbers of categorical counting, and the difference among groups and between any other two groups is analyzed by χ^2^ test. All data were analyzed with SPSS 12.0 (SPSS, Chicago, IL, United States). A *p*-value less than 0.05 is considered to be statistically significant.

## Results

### Characteristics of Bulk-Al and AlNP

**FIGURE 2:**
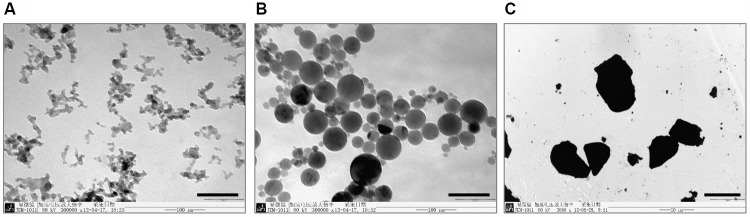
Morphology of AlNP and bulk-Al under a transmission electron microscope (TEM). To observe the morphology of and measure their particle size, AlNP and bulk-Al particles were characterized under a TEM. **(A)** AlNP of 13 nm particle size (bar = 100 nm). **(B)** AlNP of 50 nm particle size (bar = 100 nm). **(C)** Bulk-Al of 10 μm particle size (bar = 10 μm).

The primary characterization of particles was observed under TEM (**Figure 2**). Since NPs often form agglomerates in a solution, the size of the NPs and their agglomerates were estimated by Image-pro plus software. The TEM images in **Figure 2** display AlNP at 13 nm (**Figure 2A**), 50 nm (**Figure 2B**), and bulk-Al (**Figure 2C**) size in both agglomerated and non-agglomerated states. The particle size was distributed over a wide range due to differences in characteristics such as aggregation, mean diameters, and zeta potentials (**Table 1**).

**Table 1:** Particle-size and potential of AlNP and bulk-Al.

Subjects	Characteristics	
	Mean particle size (nm)	Zeta potential (mV)
13 nm AlNP	20.9 ± 9.5	49.4 ± 2.2
50 nm AlNP	112.4 ± 24.5	44.3 ± 9.1
Bulk-Al (10 μm)	8.592 ± 1.1 (μm)	

### Newborn Pups Delivered by AlNP-Treated Female Mice Had Lower BW and Higher Al Content in the Hippocampus

**FIGURE 3:**
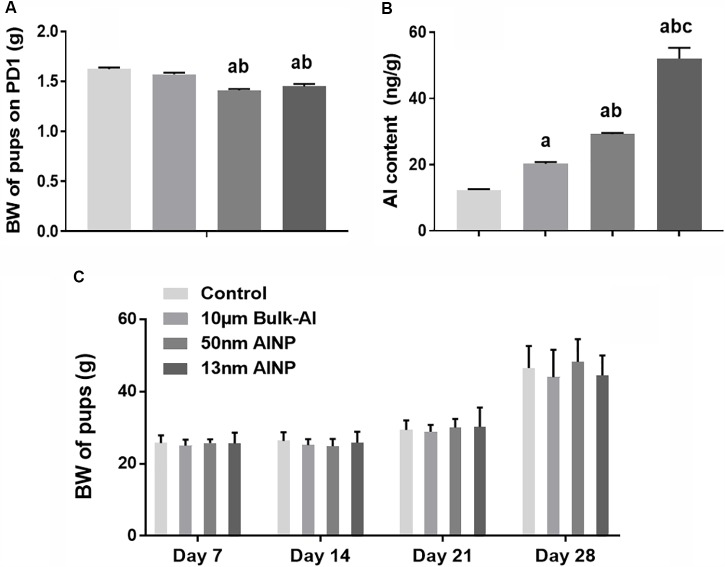
Body weight (BW) and Al contents in the newborn pups delivered by AlNP-treated female mice. To evaluate the accumulation of Al in the newborn brains, Al contents were measured using graphite furnace atomic absorption spectrometry (GFAAS). BW of newborns was used to assess the general impact of AlNP and bulk-Al particles on their next generations. The BW of PD1 newborns was significantly lower in AlNP-treated groups compared with the controls **(A)**. Al contents in the hippocampus of PD1 newborns significantly increased in bulk-Al vs. control, AlNP vs. bulk-Al and control, and 13 vs. 50 nm AlNP-treated mice **(B)**. The difference of BW in treated mice was not significant among groups though BW within each group increased by days **(C)**. a: *p* < 0.05, compared with controls; b: *p* < 0.05, compared with bulk-Al group; and c: *p* < 0.05, compared with 50 nm AlNP group.

The consumption of food and water appeared normal for all treated female mice. However, the newborn pups delivered by AlNP-treated mice had significantly lower BW on PD1 compared with controls [*F*_(3,188)_ = 30.14, *p* < 0.0001; **Figure 3A**]. Al contents in the hippocampus of PD1 pups delivered by AlNP-treated mice were significantly higher compared with those by controls [*F*_(3,44)_= 106.1, *p* < 0.0001]. There was a significant decrease in the birth weight of pups delivered by female mice treated with both 50 and 13 nm AlNP compared with those treated with bulk-Al and normal saline controls (bulk-Al vs. control, *p* = 0.0109; 13 and 50 nm AlNP vs. bulk-Al and control, *p* < 0.0001; 13 vs. 50 nm AlNP, *p* < 0.0001; **Figure 3B**). Weight gain of pups in bulk-Al and AlNP groups significantly increased from PD4 to PD7 to PD14 and to PD28 [*F*_(4,187)_= 116.9, *p* < 0.0001], but comparison of the actual body weight of pups at various weight days had no significant difference among groups [*F*_(3,188)_= 0.5364, *p* = 0.6587; **Figure 3C**].

### Newborn Pups Delivered by AlNP-Treated Female Mice Had Lower Physical Values

Ear pinna structure, appearance of teeth, and eye opening of the newborn pups were recorded in **Table 2**. The time needed for ear pinna opening among groups was significantly different [*F*_(3,188)_= 17.69, *p* < 0.0001]; ear pinna opening was delayed significantly in bulk-Al and AlNP (13 and 50 nm) groups compared with controls (*p* < 0.01). Further, AlNP (13 or 50 nm) groups were significantly delayed compared to bulk-Al group (*p* < 0.01). Similarly, the time needed for teeth appearance among groups was significantly different [*F*_(3,188)_= 85.64, *p* < 0.0001]; teeth appearance was delayed significantly in bulk-Al and AlNP (13 or 50 nm) groups compared with controls (*p* < 0.01). Furthermore, pups delivered by female mice treated with 13 nm AlNP were significantly delayed compared with bulk-Al and 50 nm AlNP groups (*p* < 0.01). Additionally, the time needed for eye opening was significantly different among groups [*F*_(3,188)_= 7.42, *p* < 0.0001], which was significantly delayed in AlNP (13 or 50 nm) groups compared with control and bulk-Al groups (*p* < 0.01), the 13 nm AlNP group displayed a greater delay in eye opening compared with 50 nm AlNP group (*p* < 0.01).

**Table 2:** Physical values in newborns delivered by AlNP-treated female mice (day).

Groups	*N*	Ear pinna open	Teeth appearance	Eyes open
Control	48	4.38 ± 0.49	10.33 ± 0.47	15.04 ± 0.69
Bulk-Al	48	4.83 ± 0.64^a^	11.11 ± 0.32^a^	15.17 ± 0.51
50 nm AlNP	48	5.15 ± 0.54^ab^	11.15 ± 0.37^a^	15.31 ± 0.84^a^
13 nm AlNP	48	5.11 ± 0.71^ab^	11.78 ± 0.61^abc^	15.69 ± 0.82^ab^

*^a^p < 0.05, compared with controls; ^b^p < 0.05, compared with bulk-Al group; and ^c^p < 0.05, compared with 50 nm AlNP group.*

### Offspring Delivered by AlNP-Treated Female Mice Had Lower Level of Neuromotor Maturation

Neuromotor maturation of pups was evaluated and the results are shown in **Table 3**. The number of righting reflex positive pups was significantly different among groups at PD4 (χ^2^= 2.856, *p* = 0.048), but no significance was found at PD7 (χ^2^= 1.644, *p* = 0.067). Compared with controls, the 13 nm AlNP group demonstrated lower righting reflexes (*p* < 0.05), while no significance was found in bulk-Al and 50 nm AlNP groups (*p* > 0.05). The number of cliff avoidance positive pups was significantly different among treated groups on PD4 (χ^2^= 4.397, *p* = 0.028) and PD7 (χ^2^= 6.12, *p* < 0.001). Compared with controls, the bulk-Al (*p* < 0.01, *p* < 0.05), 50 nm (*p* < 0.01, *p* < 0.05), and 13 nm (*p* < 0.05, *p* < 0.05) AlNP groups had fewer cliff avoidance positive mice on PD4 and PD7.

**Table 3:** Reflexing and sensation functions of the offspring delivered by AlNP-treated female mice.

Groups	Righting reflex			Cliff avoidance	
	*N*	PD4	PD7	PD4	PD7
Control	48	21	37	37	39
Bulk-Al	48	13	20	16^b^	21^a^
50 nm AlNP	48	16	34	30^b^	37^a^
13 nm AlNP	48	12^a^	29	26^a^	27^a^

*Compared with controls, ^a^p < 0.05 and ^b^p < 0.01.*

The effect of AlNP treatment on neuromotor development was evaluated on PD12 and PD14 by testing forelimb grip strength and endurance (**Table 4**). Effects of particle sizes were observed with significant differences in forelimb grip strength among groups [*F*_(3,188)_= 36.59, *p* < 0.0001]. On PD12, 50 and 13 nm AlNP groups had significantly lower forelimb grip strength compared with control and bulk-Al groups (*p* < 0.01), but no significant difference was found between 50 and 13 nm AlNP groups (*p* > 0.05). On PD14, bulk-Al, 50 nm, and 13 nm AlNP-treated mice had significantly lower forelimb grip strength compared with controls (*p* < 0.01). Furthermore, the 13 nm AlNP group demonstrated significantly lower forelimb grip strength compared with bulk-Al and 50 nm AlNP groups (*p* < 0.01), but no significant difference between bulk-Al and 50 nm AlNP groups was detected (*p* > 0.05).

**Table 4:** Forelimb grip strength in the offspring delivered by AlNP-treated female mice.

Groups	*N*	Endurance duration (s)	
		PD12	PD14
Control	48	14.80 ± 3.01	20.40 ± 2.95
10 μm Bulk-Al	48	14.77 ± 3.58	16.86 ± 1.82^a^
50 nm AlNP	48	11.28 ± 2.11^ab^	17.04 ± 2.25^a^
13 nm AlNP	48	12.13 ± 3.20^ab^	13.67 ± 2.23^abc^

*^a^p < 0.05, compared with controls; ^b^p < 0.05, compared with bulk-Al group; and ^c^p < 0.05, compared with 50 nm AlNP group.*

### Adolescents Delivered by AlNP-Treated Female Mice Displayed Greater Anxiety-Like Behavior

**FIGURE 4:**
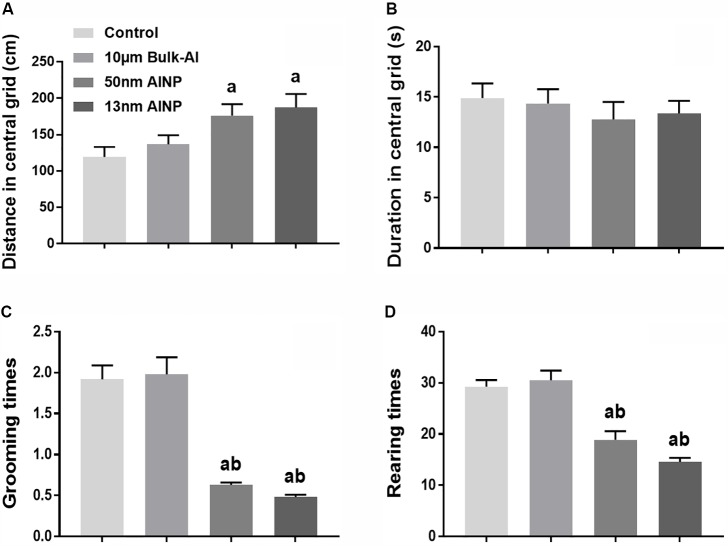
Anxiety scale of the adolescents delivered by AlNP-treated female mice. Openfield test was used to determine anxiety in the adolescents delivered by AlNP-treated female mice. The results are shown as distance in central grid **(A)**, duration in central grid **(B)**, grooming times **(C)**, and rearing times **(D)**. a: *p* < 0.05, compared with controls and b: *p* < 0.05, compared with bulk-Al group.

Neurobehavioral performance was evaluated by anxiety, learning, and memory in 1-month-old adolescents by Openfield and MWM tests (**Figure 4**). For distance in the central grids of Openfield test (**Figure 4A**), significant difference was found among groups [*F*_(3,44)_ = 4.554, *p* = 0.0061]. The distance in central grids traveled by pups in the AlNP (50 and 13 nm) groups was significantly higher than that in control and bulk-Al treated groups (*p* = 0.047 and 0.0116), but no statistical significance was found between bulk-Al and controls (*p* = 0.8425). For duration in central grid (**Figure 4B**), no significant difference was found among groups [*F*_(3,44)_ = 0.4194, *p* = 0.7396]. For grooming times (**Figure 4C**), a significant difference was found among groups [*F*_(3,44)_ = 35.83, *p* < 0.0001], though no significant difference was found between bulk-Al treated group and controls (*p* = 0.9891). However, there was a significant decrease in the 50 and 13 nm AlNP-treated groups (both *p* < 0.0001) compared with bulk-Al and controls. In addition, no significant difference was found between 50 and 13 nm AlNP-treated groups (*p* = 0.8607). For rearing times (**Figure 4D**), a significant difference was found among groups [*F*_(3,44)_= 27.70, *p* < 0.0001], though no significant difference was found between bulk-Al treated group and controls (*p* = 0.9329). However, there was a significant decrease in rearing times of 50 and 13 nm AlNP-treated groups (both *p* < 0.0001) when compared with bulk-Al group and controls, but no significance between the AlNP-treated groups (50 and 13 nm; *p* = 0.1816).

### Adolescents Delivered by AlNP-Treated Female Mice Had Worse Neurobehavioral Performance

**FIGURE 5:**
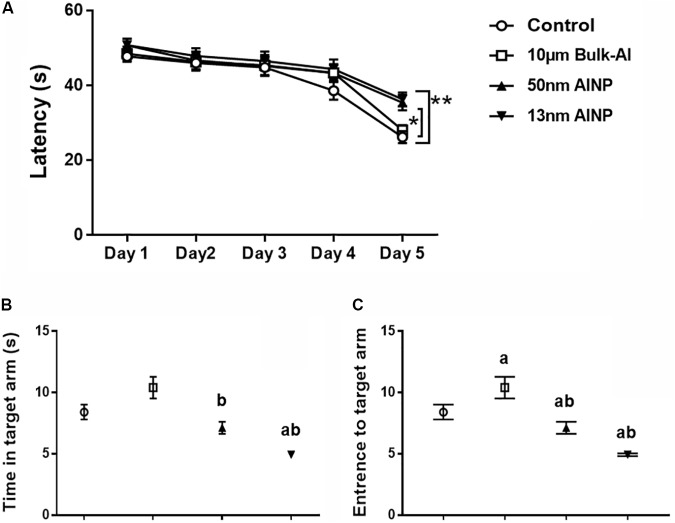
Learning and memory performance in the adolescents delivered by AlNP-treated female mice. Morris water maze was used to assess learning and memory performance in the offspring delivered by AlNP-treated female mice. Learning **(A)** and memory **(B,C)** performance of the offspring delivered by AlNP-treated female mice was displayed. a: *p* < 0.01 compared with controls and b: *p* < 0.01, compared with bulk-Al group.

Morris water maze task was used to determine learning and memory performance in adolescents delivered by AlNP-treated female mice (**Figure 5**). The adolescents demonstrated a significant decrease (**Figure 5A**) in learning performance within 5-day training [*F*_(4,235)_= 42.93, *p* < 0.0001] with particle-size effects among groups [*F*_(4,235)_= 4.452, *p* = 0.0044]. No significant difference was found between bulk-Al treaded group and controls (*p* = 0.6223), but a significant decrease in learning was found in 50 (*p* = 0.0410) and 13 nm (*p* = 0.0053) AlNP-treated groups compared with controls. However, no significant difference was found between bulk-Al group and 50 (*p* = 0.4691) and 13 nm (*p* = 0.1473) AlNP-treated groups, nor between the 50 and 13 nm AlNP-treated groups (*p* = 0.9064).

Memory profiles also differed in the amount of time spent in the target arm (**Figure 5B**) among groups [*F*_(3,44)_= 14.92, *p* < 0.0001]. No significant difference was found compared bulk-Al (*p* = 0.0942) and 50 nm AlNP-treated groups (*p* = 0.4299), but adolescents in 13 nm AlNP-treated group spent less time in the target arm (*p* = 0.0006) compared with controls. Compared with bulk-Al, adolescents in 50 (*p* = 0.0.0014) and 13 nm (*p* < 0.0001) AlNP-treated groups spent less time in target arm but no significance was found between 50 and 13 nm AlNP-treated groups (*p* = 0.0529). There was also a significant decrease in the number of entries into the target arm (**Figure 5C**) among groups [*F*_(3,44)_ = 60.29, *p* < 0.0001]. Compared with controls, adolescents in bulk-Al (*p* < 0.0001) and 50 and 13 nm AlNP-treated groups (both *p* < 0.0001) all had less entries to the target arm, while those in 50 and 13 nm AlNP-treated groups had much less entries compared with that in bulk-Al group (**Figure 5**, both *p* < 0.0001), though no significance was found between 50 and 13 nm AlNP-treated groups (*p* = 0.9997).

### Oxidative Stress in Adolescents Delivered by AlNP-Treated Female Mice

**FIGURE 6:**
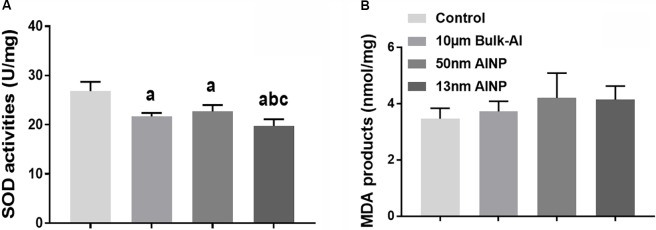
Oxidative stress in the cerebral cortex of the adolescents delivered by AlNP-treated female mice. To explore the mechanism involved in the neurobehavioral deficits, oxidative stress levels in the cerebral cortex were quantified. SOD activity **(A)** and MDA products **(B)** as measurements of oxidative stress were examined. There was a significant difference in both SOD activity and MDA products among groups. a: *p* < 0.05, compared with controls; b: *p* < 0.05, compared with bulk-Al group; and c: *p* < 0.05, compared with 50 nm AlNP group.

Superoxide dismutase activity and MDA products in the cerebral cortex were measured to evaluate oxidative stress in adolescents delivered by AlNP-treated female mice (**Figure 6**). SOD activity was significantly different among groups [**Figure 6A**, *F*_(3,44)_ = 38.5, *p* < 0.0001]. Compared to controls, SOD activity in bulk-Al and AlNP (50 and 13 nm) treated groups was significantly lower (*p* < 0.0001 for all). No significance was found between bulk-Al and 50 nm AlNP-treated groups (*p* = 0.4365), while SOD activity in 13 nm AlNP-treated mice was significantly lower compared with those in bulk-Al and 50 nm AlNP-treated groups (*p* = 0.0387 and 0.0008). For MDA products, though a significant difference in MDA products in the cerebral cortex among groups was found [**Figure 6B**, *F*_(3,44)_= 3.121, *p* = 0.0417], no significant difference (*p* > 0.05) was found between any two groups (*p* > 0.05).

### Neurotransmitter Levels in the Cerebral Cortex of Adolescents Delivered by AlNP-Treated Female Mice

**FIGURE 7:**
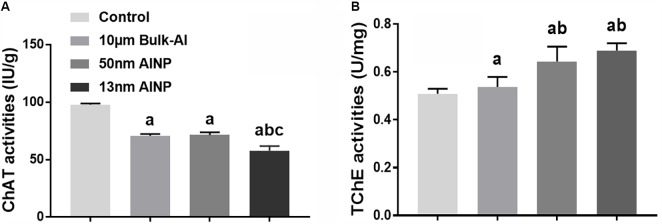
Neurotransmitter levels in cerebral cortex of adolescents delivered by AlNP-treated female mice. To explore the mechanism involved in the neurobehavioral deficits, neurotransmitter levels in the cerebral cortex were quantified. Choline acetyltransferase **(A)** and total cholinesterase activities **(B)** were examined. a: *p* < 0.05, compared with controls; b: *p* < 0.05, compared with bulk-Al group; and c: *p* < 0.05, compared with 50 nm AlNP group.

Neurotransmitter enzyme levels of ChAT and TChE were measured in the cerebral cortex of adolescents delivered by AlNP-treated female mice (**Figure 7**). ChAT activity was significantly different among groups [**Figure 7A**, *F*_(3,44)_ = 368.3, *p* < 0.0001]. Compared to controls, ChAT activity in bulk-Al and AlNP-treated groups (50 and 13 nm) was significantly decreased (*p* < 0.0001 for all). Although ChAT activity in bulk-Al vs. 50 nm AlNP-treated mice did not differ (*p* = 0.9653), mice in 13 nm AlNP-treated group had significantly lower levels of ChAT compared to bulk-Al and 50 nm AlNP-treated mice (*p* < 0.0001 both). For TchE activity, a significant difference among groups was found [**Figure 7B**, *F*_(3,44)_ = 34.6, *p* < 0.0001]. Though no significance was found between bulk-Al treated group and controls (*p* = 0.5440), the TChE activity in AlNP (50 and 13 nm) treated groups was significantly higher compared with bulk-Al treated group and controls (*p* = 0.0001 and *p* < 0.0001). There was no significance found between 50 and 13 nm AlNP groups on TChE activity (*p* = 0.1460).

## Discussion

The developing nervous system is especially vulnerable to toxic chemical exposures (Rodier, 1995; Bondy and Campbell, 2005), and major windows of developmental vulnerability occur *in utero* and during infancy and early childhood (Rice and Barone, 2000). During these sensitive life stages, exposure to chemicals, even at low concentrations, can cause permanent brain injury that can manifest as functional impairments or disease at any point in the human lifespan—from early infancy to old age. With the rapid development of nanotechnology, the likelihood of environmental exposure to NPs has significantly increased either actively or passively (Service, 2004). Although BBB and blood–placental barrier can provide some protection against the entry of external products into the CNS during fetal development and early infancy, exposure to untested NPs should not be presumed safe. As new products with distinguished chemical and physical characteristics differ from their bulk counterparts, NPs in existing use and all new products must therefore be tested for developmental neurotoxicity (Grandjean and Landrigan, 2014).

The placenta is a multifunctional organ constituting the barrier between maternal and fetal tissues. Research has shown that NPs can cross the placental barrier (Keelan, 2011; Muoth et al., 2016), and there is increasing evidence that the extent of transfer is dependent on particle characteristics and functionalization (Yang et al., 2012; Tian et al., 2013). It is reported that exposure of pregnant mice to different NPs induces pregnancy complications or damage to the fetus (e.g., to silver NPs, titanium dioxide, silicon dioxide, and carbon nanotubes) (Shimizu et al., 2009; Hougaard et al., 2010; Pietroiusti et al., 2011; Yamashita et al., 2011; Campagnolo et al., 2013, 2017; Huang et al., 2014; Qi et al., 2014). Given the high susceptibility of the CNS to external insults during the developmental period, we address specific concerns on neurodevelopmental toxicity effects of AlNP in the present study.

Since developmental events are intertwined in the reproductive process, effects on developmental toxicity may be detected in developmental combined reproductive studies. Evaluation of the developmental effects in the present study is based on the National Toxicity Program Criteria from the U.S. Department of Health and Human Services for Levels of Evidence for Developmental Toxicity (Foster, 2009). A standard Reproductive/Developmental Toxicity Screening Assay (Organization for Economic Cooperation and Development [OECD], 2016) is used in female mice dosed with AlNP for 2 weeks prior to mating, during mating, and throughout gestation until the pups were born. Compared with developmental toxicological experiments that treatments are designed to start from the first day of mating, our experimental design of treatment starting from 2 weeks before mating considers the potential toxicity *in utero* to the female and toxicological consequences in the offspring.

First, the BW of newborn developing pups delivered by AlNP-treated female mice decreased significantly while Al contents in the hippocampus of PD1 newborns were increased (**Figure 3**). It was found that BW of the newborns on PD1 was significantly lower in pups delivered by AlNP-treated female mice. Moreover, PD1 newborns in AlNP-treated groups had significant higher contents of Al element in the hippocampus, indicating that AlNP can enter the hippocampus of the embryos and result in greater neuronal damage due to known Al neurotoxicity. Furthermore, neurodevelopmental deficits persisted into adolescents even though their lower BW was reconciled on the following PD7–PD28.

Second, AlNP administration via nasal drip in pregnant female mice affected the physical developmental landmarks in their offspring (**Table 2**). Development of morphological indicators including ear pinna structure, appearance of teeth, and eye opening of the newborn pups delivered by AlNP-treated female mice was significantly delayed compared to the controls. In particular, the smaller sized AlNP (13 nm) caused a greater delay in developmental characteristics compared to 50 nm AlNP-treated group, suggesting possible interference in molecular pathways involved in physical growth and development.

Third, AlNP suppressed CNS maturation in the offspring. Normal neuromotor responses, such as righting reflex and cliff avoidance, were reduced significantly (**Table 3**) and the endurance test demonstrated worse neuromuscular skeletal development in the offspring delivered by AlNP-treated female mice (**Table 4**). These findings suggest AlNP-induced defects in maturation of the offspring’s CNS when AlNP are prenatally exposed to the developing fetus *in utero*. It is possible that continuous exposure to AlNP during fetal development *in utero* has a cumulative toxic effect in the developing brains. Persisting neurodevelopmental deficits are indicative of potentially irreversible neural damage or improper neurodevelopment.

Finally, adolescent offspring in AlNP groups demonstrated greater anxiety-like behavior (**Figure 4**) and decreased learning and memory performance (**Figure 5**), indicating that prenatal exposure of AlNP in female mice during pregnancy can have long-lasting changes in neurocognitive function of the offspring which continues through adolescence. Evidence of elevated oxidative stress (**Figure 6**) and decreased neurotransmitter levels (**Figure 7**) in cerebral cortex of the adolescents suggests that prenatal AlNP exposure can induce brain dysfunction in adolescents by promoting oxidative stress accumulation while suppressing availability of neurotransmitters. In fact, studies have demonstrated repeatedly that AlNP can induce ROS and reduce neurotransmitter content in brain (Sanchez-Iglesias et al., 2009; Fu et al., 2014; Simko and Mattsson, 2014; Shah et al., 2015).

Therefore, it is suggested in the present study that AlNP exposure of female mice during pregnancy can jeopardize the proper development of the brain in offspring, which is one of the most susceptible target organs during early perinatal period. It also appears that AlNP-induced neurodevelopmental deficits can persist into adulthood, as shown by altered anxiety-like behavior and cognitive function. An important line of evidence in this study was that oxidative stress and neurotransmitter enzyme levels in offspring were altered by AlNP exposure of female mice during pregnancy, although further work is necessary to determine whether these findings precede neurobehavioral outcomes and lead to neurodevelopmental deficits or whether altered neurophysiology brought by perinatal exposure to AlNP leads to elevated oxidative stress and reduced neurotransmitter contents. Further studies to examine the relationship between AlNP and neurodevelopment are needed to investigate associated mechanisms and potential therapeutic strategies during the perinatal period, before permanent changes to the brain have occurred.

## Author Contributions

QZ takes charge of experimental design, implementation, results, discussion, manuscript revision, and agrees to be accountable for all aspects of the work. YD, HL, and XW take charge of experimental data acquisition, analysis, manuscript drafting, and agree to be accountable for the animal experiments. KH takes charge of experimental data interpretation, manuscript drafting, and agrees to be accountable for the data interpretation. FG takes charge of experimental data acquisition of nanoparticles, analysis, manuscript revision, and agrees to be accountable for the nanoparticle characteristics experiments. TM and JD take charge of experimental data interpretation, manuscript drafting and revision, and agree to be accountable for the accuracy of interpreting and discussion of the experiments. QN takes charge of experimental design, revision of the manuscript, and agrees to be accountable for the aspects of experiments design and concepts. All authors approved the final version of the manuscript to be published.

## Conflict of Interest Statement

The authors declare that the research was conducted in the absence of any commercial or financial relationships that could be construed as a potential conflict of interest.
